# Impact of temporomandibular disorder symptoms among 15-year-old girls

**DOI:** 10.2340/aos.v83.41113

**Published:** 2024-08-23

**Authors:** Christina Mejersjö, Eva-Karin Bergström, Curt Hagquist, Anders Wänman

**Affiliations:** a Sahlgrenska Academy of Gothenburg University and the Public Dental Service of Västra Götaland, Gothenburg, Sweden; b Department of Cariology, Institute of Odontology, Sahlgrenska Academy, Gothenburg University, Gothenburg, Sweden; c Department of Education and Special Education, Gothenburg University, Gothenburg, Sweden; d Department of Clinical Oral Physiology, Umeå University, Umeå, Sweden

**Keywords:** temporomandibular disorders, adolescents, impact, general health, physical activity

## Abstract

**Objective:**

Many adolescents, especially girls, report temporomandibular disorder (TMD) symptoms that may impact their daily life.

**Methods:**

At 19 different schools participating in the preventive program with dental nurses of FRAMM (Fluoride, Advise, Arena, Motivation, Food), at the Västra Götaland Region in Sweden, 15-year-old girls were invited to a cohort study about symptoms of TMD that also included headaches. Three hundred twenty-nine girls attended the study and answered a questionnaire regarding TMD symptoms and their consequences such as sick leave from school and consumption of analgesics. The girls were asked about the symptom’s influence on their daily life, about their general health, use of regular medication, physical activity, and they answered the PHQ4 regarding experienced symptoms of anxiety and depression.

**Results:**

There was a significant correlation between TMD symptoms and sick leave with 31% of the girls having stayed home due to symptoms. Of the girls who answered affirmative in the screening questions (3QTMD), nearly half the group had stayed at home due to their symptoms, 24% had consulted a physician, 42% had used analgesics weekly and 59% reported that they felt the symptoms negatively affected their school performance. The girls who had regular medication had more TMD symptoms. Anxiety and depression were associated with TMD symptoms.

**Conclusion:**

The study showed that TMD symptoms had a negative impact on the 15-year-old girls’ daily life resulting in sick leave from school, consumption of analgesics, and experiences of negative impacts on their behavior and performance at school.

## Introduction

Temporomandibular disorder (TMD) symptoms are prevalent in the population, including among adolescents [1–3], and comprise several different symptoms involving the jaw muscles, the temporomandibular joints, and related structures [4]. Symptoms like orofacial pain and impaired function when eating, yawning, and talking are frequently reported. Also reported are temporomandibular joint noise and impaired jaw mobility. Headache in the temples and forehead regions is often associated with TMD symptoms [5, 6], and these symptoms may generate concerns and worries among the girls [7]. A previous study reported that nearly one-third of adolescent girls had school absences as well as analgesic consumption because of TMD-pain [8]. Easy access to internet-based information can both impair and improve how these symptoms are comprehended by young people. Therefore, it is important to carefully examine how these symptoms affect the young population.

The etiology of the symptoms is complex, multifactorial, and best understood in a bio-psycho-social context. Some known risk factors for developing TMD symptoms are related to behaviors such as grinding and clenching teeth, pain in other parts of the body, and female gender [9, 10]. The onset of TMD symptoms appears to be in proximity to the onset of puberty [1].

Studies have found a prevalence of TMD of up to 30% among adolescents [2, 11, 12], and the symptoms at this age are mostly regarded to have a muscular genesis [12, 13]. Among adult women with TMD symptoms, about half reported a negative impact on their life [14]. In a review of the impact of TMD symptoms in adults, nearly everyone with symptoms expressed a negative impact on their quality of life, and the impact was increased with more severe symptoms [15]. TMD symptoms have been associated with anxiety, depression, and perceived stress [16].

TMD-pain symptoms have been associated to lead to substantial consequences for many aspects of life for a teenage TMD patient [12, 17]. In adolescents, it was shown that TMD-pain symptoms were associated with anxiety, depression, and somatization [18], and TMD symptoms were associated with impaired general health [19, 20]. However, little is known about the impact of TMD pain and dysfunction on the non-patient lower teenage population.

Physical activity is recommended to reduce pain and support general relaxation [21], and physical training has been used with positive results for the treatment of some pain conditions like fibromyalgia [22]. The need for any treatment due to TMD symptoms has been estimated at 15%–16% among nonpatients’ samples [3, 23]. Researchers have advocated that the dental health service must pay attention to TMD symptoms and make greater efforts to manage these symptoms [13]. Prophylactic information was found to possibly influence the symptoms [24].

The aim of the study was to investigate the impact and consequences of TMD symptoms and headache on the daily life activities among a 15-year-old girl population, and if the quantity of physical activity influenced the degree of TMD symptoms.

## Materials and methods

In 21 elementary schools in the Western Sweden, the management was contacted and was asked about participating in a study of teenaged girls and TMD. Nineteen schools accepted to participate. The schools had been selected in the scheme of the dental nurses’ school visits with the intent to obtain a mixture of schools of different sizes and located in different social environments, in both cities and rural areas, and included both municipal and independent schools. Girls in the middle schools (upper level of compulsory school), 15 years of age, were invited to participate and were informed about the study both orally and in writing. Their guardians were also informed and they could choose to refuse participation. All the girls in a class were invited, participation was voluntary, and no parents refused their girl in participating. At the nurses visit at a school, there were always one or two girls absent (e.g. from sick leave). A total of 329 girls attended the study, which was regarded as a representative group of 15-year-old girls of the district. The girls were only identified by name and class. The study was approved by the regional Committee of Ethics in Göteborg, Sweden (no 457-18).

The girls themselves answered a questionnaire regarding TMD symptoms and headaches, which was a combination of previously used parts of questionnaires for TMD [3, 24] and for the ‘Youth in Värmland’ questionnaire [25]. The questionnaire was answered at the schools from March to May in 2021 and in the same monthly period in 2022.

Reported symptoms were asked for, six different symptoms and the frequency: feeling tired/stiff in the jaws, temporomandibular joint clicking, headache, pain in the orofacial region, difficulty to open wide, or the jaw being locked (0 = never or seldom, 1 = 1–2 times/month, 2 = 1–2 times/week, 3 = 3–4 times/week, and 4 = daily). To get an idea of a girl’s total frequency of reported TMD symptoms and headaches, a symptom index was created by adding the frequencies of each of the six symptom resulting in a total of 0–24 points for the symptom index.

The questionnaire also included the three screening questions for TMD (3QTMD) yes/no [26], a screening instrument regarding symptoms once a week or more often and not including all of the different symptoms asked for separately:

Q1: ‘Do you have pain in your temple, face, jaw, or jaw joint once a week or more?’

Q2: ‘Do you have pain when you open your mouth or chew once a week or more?’

Q3: ‘Does your jaw lock or become stuck once a week or more?’

The girls were asked to describe the influence that the symptoms had on their relationship with parents, friends, school, and activities in their spare time. They also reported the severity of the symptoms estimated on a visual analogue scale (VAS), whether the symptoms had led to sick leave from school and use of analgesics and if a physician had been consulted due to the symptoms.

The girls were also asked to complete the PHQ4 [27], which is a four-item patient health questionnaire for anxiety and depression that gives a score of 0–12. The scores are rated as normal (0–2), mild (3–5), moderate (6–8), and severe (9–12).

The girls were also asked to estimate their self-perceived general health 1–5 (1 = excellent, 2 = very good, 3 = good, 4 = fairly good, 5 = poor) and to describe any known medical diagnosis and medication used regularly.

Finally, they were asked to estimate their amount and type of physical activity outside school (1 = almost no physical activity, 2 = sometimes a walk or cycling, 3 = light exercise, in total 2 h/w, 4 = exercise, in total at least 3 h/w, 5 = hard exercise or competition several times/w, at least 2 h on each occasion).

Statistical processing and analyses of the questionnaires were processed using the SPSS 20 software (SPSS Inc., Chicago, IL). The Chi 2 test was used for comparison between reported symptoms and factors like general health, use of analgesics, sick leave, depression and anxiety, physical activity, and the impact of the TMD symptoms. The Mann–Whitney test was used for comparing groups. Statistically significant differences and correlations of a strength of *p* ≤ 0.05 are presented.

## Results

### Sick leave and medical advice

Thirty one percent reported any sick leave from school due to TMD symptoms and/or headaches during the preceding 3 months. There was a significant association between reported sick leave and one or more affirmative answers to the 3QTMD, as well as with the symptom index (*p* < 0.001). Medical advice related to complaints from the jaw and/or head had been sought by 24% of those with an affirmative answer to the 3QTMD (Table 1).

**Table 1:** Prevalence of reported sick leave from school, utilization of medical care, and use of analgesics due to TMD symptoms, self-perceived general health, PHQ4 score, VAS, and subjective symptom index during the preceding 3 months, among 329 15-year-old girls. The distribution for included variables between those who stated a ‘no’ (n = 217) or a ‘yes’ (n = 112) to one or more of the 3QTMD screening questions [22], and p-value is presented.

Variables		Total %	No to 3QTMD (%)	Yes to 3QTMD (%)	*p*
Sick leave from school	1–2 days	21	17	29	<0.001
	3–4 days	7	5	11	
	> 5 days	3	1	6	
Sought medical care due to the symptoms		12	0	24	
Use of analgesics	1–2 times/w	21	18	27	0.001
	3–4 times/w	6	4	11	
	Daily	2	1	5	
Self-perceived health	Excellent – very good	41	48	34	0.002
	Good – fairly good	56	50	63	
	Poor	3	2	3	
Anxiety and depression (PHQ4 score)	Moderate	21	16	29	<0.001
	Severe	9	3	14	
VAS (mean and SD)			11.3 (16.5)	41.5 (21.2)	
Subjective symptom index (mean)			2.5	7.9	<0.001

3QTMD, three screening questions for TMD.

### Symptom intensity

The mean VAS value for the group with one or more affirmative answers to the 3QTMD was 41.5 and for those with a negative answer was 11.3. The proportion of girls with a VAS score ≥ 50 was 40.5% for the group with an affirmative answer to the 3QTMD, and was 2.9% for the group answering ‘no’. The mean value of the symptom index was 7.9 for the group with an affirmative answer to the 3QTMD and was 2.5 for the group answering ‘no’ (Table 1).

### Analgesics

Due to symptoms of the jaw and head, 44% of all the girls had sometimes used analgesics, 30% had used analgesics once a week or more. Among those with an affirmative answer to the 3QTMD, 42% had used analgesics once a week or more and 4.5% reported daily use (Table 1). The use of analgesics was associated with sick leave from school due to symptoms from the jaw and/or head (*p* < 0.001).

### Impact of the symptoms

The symptoms of TMD and/or headache influenced the girls’ daily life with behavior at school and the ability to perform schoolwork as the most affected aspects according to the girls´ estimation. All five situations inquired about in the questionnaire were significantly correlated to the subjective index (*p* < 0.005) (Table 2). Girls with one or more affirmative answers to 3QTMD reported a significantly more negative impact compared with those who gave a ‘no’ answer (*p* < 0.001); 59% reported a negative impact on their behavior at school (Table 2).

**Table 2:** Percentage distribution of reported negative influence on the daily life from TMD symptoms and/or headache among 15-year-old girls with any reported TMD symptoms and for those answering ‘no’ and those answering ‘yes’ to one or more of the 3QTMD screening questions [22].

Negative influence regarding	Any TMD symptom (%) *n* = 214	No to 3QTMD (%) *n* = 173	Yes to 3QTMD (%) *n* = 112
Relations to parents	15	10	23
Relations to friends	16	10	21
School work	44	34	59
Behavior at school	24	26	35
Leisure time activities	25	20	32

TMD: temporomandibular disorder; 3QTMD: three screening questions for TMD.

### General health

Impaired self-perceived health was associated with the symptom index score (*p* < 0.001). Regular medication was associated with symptoms according to the 3QTMD (Table 1) as well as with a higher symptom index score compared with those without medication (*p* < 0.001). Also, having a regular medication was associated with a higher index score on the PHQ4 (*p* = 0.003).

### Experienced symptoms of anxiety and depression

The PHQ4 showed a significantly higher index score for those with an affirmative answer to the 3QTMD as well as for the symptom index, compared to the whole group of 15-year-old girls (*p* < 0.001) (Table 1). The PHQ4 score significantly correlated with the screening questions about pain and pain on opening the mouth or chewing, as well as to the reported symptoms of orofacial pain and of headache (*p* < 0.001).

A negative impact due to the symptoms was found regarding the girls’ relationships with friends (*p* < 0.001) and the perception of their behavior at school (*p* = 0.008) but not with absence from school due to sickness (Table 2).

### Physical activity

The girls’ reported quantity of physical activity was not associated with the degree of TMD symptoms. However, those with a high level of physical activity and training reported more headache (*p* < 0.001).

## Discussion

The study confirms that TMD symptoms and headaches have a strong negative impact on the daily life of teenage girls pertaining to their behavior and their performance at school, as well as to their use of analgesics and sick leave from school. The finding that the symptoms negatively influence their social activities is in contrast with Al-Khonti who found that TMD-pain does not seem to affect social activities [18]. More than one-third of the girls estimated their TMD symptoms and/or headache as high on the VAS scale. This confirms the results from other studies on the impact of TMD symptoms and headache [2, 3, 11] and emphasizes the practical consequences for the 15-year-old girls.

The index for anxiety and depression was significantly higher among those with TMD symptoms that corresponds to the findings of other studies [17, 19, 28]; however, few previous studies regard young adolescents. The symptoms of TMD and/or headache were shown to have a negative influence on the girls’ daily life and could induce anxiety both because of the symptoms and of thoughts about the future prognosis. Even if the symptoms do not cause depression they may give rise to depressive thoughts, impair the girls’ self-confidence, and cause them to avoid social activities. About 3.2% of the girls had medication for some kind of psychiatric problems. The PHQ4 score among girls without TMD symptoms also indicated anxiety and depression but to a lesser extent.

The score for anxiety and depression was significantly associated with orofacial pain and with headache. The association between pain and anxiety and depression was also found in other studies [12, 17].

The association between TMD symptoms and self-estimated health among adults is known, and this was also seen among the 15-year-old girls. The self-estimated health status is a combination of experiences and feelings about one’s health and is of course influenced by the TMD symptoms but also by one’s mood and the degree of anxiety and depression. The association between TMD symptoms and regular medication is in accordance with what was found previously. A study of children and adolescents in Japan found that pain in the neck was associated with orofacial pain conditions and headache [19]. In another study, body pain and systemic diseases of adolescents were associated with painful TMD symptoms; this also pertained to diagnoses like bronchitis and asthma [20].

The questionnaire used in the present study was a combination of parts from previously used questionnaires for dental and youth studies. It was short enough to be answered by the girls in the short time available at schools, and it provided information about the many aspects inquired about. An index of the oral-health-related-quality of life, such as the validated and widely used Oral Health Impact Profile (OHIP5) [29], could have been used, but the OHIP5 considers oral and facial conditions and not specifically TMD. It could have complemented the requirements of the present study but could not replace the questionnaire used.

The present study deals with both TMD and headache since headache is closely associated with TMD [30]. However, not all reported headaches are associated with TMD. Migraine has been estimated to affect 8%–9% of children and adolescents [31]. It is sometimes difficult to distinguish between the diagnoses of tension headache and migraine. The difference between facial pain and headache was also difficult to describe to some of the girls, and there may have been some confusion. Pain in the temple is included in one of the screening questions of the 3QTMD, but some girls did not associate that question with headache.

Physical activity is known to reduce tension, to activate the muscles of the whole body, and to induce relaxation [21]. Our study did not find any obvious association between the amount of physical activity and the degree of TMD symptoms among the 15-year-old girls. However, girls with intense physical activity and exercise reported more headache, which complies with the finding that high-intensity physical activity is associated with more TMD pain, and thus the recommendation to choose low-intensity activity [32].

The 3QTMD [26] is a valuable instrument for screening dental patients for TMD symptoms. However, there are TMD symptoms not included in these three questions; therefore, a girl can have a negative answer to the 3QTMD but still have a score in the symptom index, like feeling tired or stiff, which could be the first symptom of overloading of the jaw. There is also a difference between 3QTMD and the symptom index regarding the frequency of the symptoms.

A weakness of our study is that the number of dropouts is not known. Some girls, although few, were not in their class when the questionnaires were answered likely due to sick absence or holiday. The study includes only reported symptoms and a clinical examination would have completed the picture, however, a previous study gives both reported symptoms and clinical signs for some of the girls [3]. A strength of our study is that the study population was rather large and was spread out geographically and socioeconomically since schools of different areas were invited to the study; this adds credibility to the results. The participating girls are considered representative for 15-year-old girls in Västra Götaland, Sweden.

## Conclusion

TMD symptoms and headache lead to many negative consequences in the daily life of young teenaged girls. The amount of physical activity showed no association with the degree of TMD symptoms.

## Data Availability

The research data associated with the paper are available from the authors Professor Anders Wänman anders.wanman@umu.se and Docent Christina Mejersjö christina.mejersjo@vgregion.se and the data in the SPSS-files can be made available from the authors on request.

## References

[1] Lövgren A, Häggman-Henrikson B, Vischer CM, Lobbezoo F, Marklund S, Wänman A. Temporomandibular pain and jaw dysfunction at different ages covering the lifespan-a population-based study. Eur J Pain. 2016;20:532–540. 10.1002/ejp.755 26311138

[2] Christidis N, Ndanshau EL, Sandberg A, Tsilingaridis. Prevalence and treatment strategies regarding temporomandibular disorders in children and adolescents – a systematic review. J Oral Rehabil. 2019;46:291–301. 10.1111/joor.12759 30586192

[3] Mejersjö C. Bergström E-K, Wenneberg B, Wänman A. TemporoMandibular Disorder symptoms in 13–15 years old females; reported symptoms and clinical signs. Int J Dent Hygiene. (Ahead of print).

[4] Okeson JP. Signs and symptoms of temporomandibular disorders. In: Okeson JP, editor. Management of temporomandibular disorders and occlusion. 7th ed. St. Louis, MO: Mosby, Elsevier Inc.; 2013, p. 129–169.

[5] Emshoff R, Bertram F, Schnabl D, Emshoff I. Association between chronic tension-type headache coexistent with chronic temporomandibular disorder pain and limitations in physical and emotional functioning: a case-control study. J Oral Facial Pain Headache. 2017;31:55–60. 10.11607/ofph.1654 28118421

[6] Nilsson IM, List T, Drangsholt M. Headache and co-morbid pains associated with TMD pain in adolescents. J Dent Res. 2013;92: 802–807. 10.1177/0022034513496255 23813050

[7] Wainwright E, Jordan A, Fisher E, Wilson C, Mullen D Madhavakkannan H. Beliefs about worry and pain amongst adolescents with and without chronic pain. J Pediatr Psychol. 2022;47:432–445. 10.1093/jpepsy/jsab109 34725707

[8] Nilsson IM, Drangsholt M, List T. Impact of temporomandibular disorder pain in adolescents: differences by age and gender. J Orofac Pain. 2009;23:115–122. 19492536

[9] Marklund S, Wänman A. Risk factors associated with incidence and persistence of signs and symptoms of temporomandibular disorders. Acta Odontol Scand. 2010;68:289–299. 10.3109/00016357.2010.494621 20528485

[10] Slade GD, Ohrbach R, Greenspan JD, et al. Painful temporomandibular disorder: decade of discovery from OPPERA studies. J Dent Res. 2016;95:1084–1092. 10.1177/0022034516653743 27339423 PMC5004239

[11] Paduano S, Bucci R, Rongo R, Silva R, Michelotti A. Prevalence of temporomandibular disorders and oral parafunctions in adolescents from public schools in Southern Italy. J Oral Rehabil. 2023;50:99–112. 30547719 10.1080/08869634.2018.1556893

[12] Restrepo C, Ortiz AM, Henao AC, Manrique R. Association between psychological factors and temporomandibular disorders in adolescents of rural and urban zones. BMC Oral Health 2021;21:140. 10.1186/s12903-021-01485-4 33743662 PMC7981971

[13] Mélou C, Sixou J L, Sinquin C, Chauvel-Lebret D. Temporomandibular disorders in children and adolescents: a review. Archives de Pédiatrie. 2023;30:335–342. 10.1016/j.arcped.2023.03.005 37147156

[14] Storm Mienna C, Wänman A. Self-reported impact on daily life activities related to temporomandibular disorders, headaches, and neck-shoulder pain among women in a Sami population living in Northern Sweden. J Orofac Pain. 2012;26:215–224. 22838006

[15] Dahlström L, Carlsson GE. Temporomandibular disorders and oral health-related quality of life. A systematic review. Acta Odontol Scand. 2010;68:80–85. 10.3109/00016350903431118 20141363

[16] Wieckiewicz M, Jenca Jr A, Seweryn P, Orzeszek S, Petrasova A, Grychowska N, et al. Determination of pain intensity, pain-related disability, anxiety, depression, and perceived stress in Polish adults with temporomandibular disorders: a prospective cohort study. Front Integr Neurosci. 2022:16:1026781. 10.3389/fnint.2022.1026781 36407294 PMC9668250

[17] Nilsson I-M, List T, Willman A. Adolescents with temporomandibular disorder pain-the living with TMD pain phenomenon. J Orofac Pain. 2011;25:107–116. 21528117

[18] Al-Khotani A, Naimi-Akbar A, Gjelset M, Albadawi E, Bello L, Hedenberg-Magnusson B, et al. The associations between psychosocial aspects and TMD-pain related aspects in children and adolescents. J Headache Pain. 2016;17:30. 10.1186/s10194-016-0622-0 27044436 PMC4820412

[19] Karibe H, Shimazu K, Okamoto A, Kawakami T, Kato Y, Warita-Naoi S. Prevalence and association of self-reported anxiety, pain, and oral parafunctional habits with temporomandibular disorders in Japanese children and adolescents: a cross-sectional survey. BMC Oral Health. 2015;15:8–12. 10.1186/1472-6831-15-8 25604542 PMC4324877

[20] do Vale Braido GV, Campi BL, Jordani PC, Fernandes G, de Godoi GonCalves DA. Temporomandibular disorder, body pain and systemic diseases: assessing their associations in adolescents. J Appl Oral Sci. 2020;28:e20190608. 10.1590/1678-7757-2019-0608 32901693 PMC7480668

[21] Mahindru A, Patil P, Agrawal V. Role of physical activity on mental health and well-being: a review. Cureus. 2023;15(1):e33475. 10.7759/cureus.33475 36756008 PMC9902068

[22] Bidonde J, Busch AJ, Schachter CL, et al. Mixed exercise training for adults with fibromyalgia. Cochrane Database Syst Rev. 2019;5:CD013340. 10.1002/14651858.CD013340. 10.1002/14651858.CD013340 31124142 PMC6931522

[23] Al-Jundi MA, John MT, Setz JM, Szentpétery A, Kuss O. Meta-analysis of treatment need for temporomandibular disorders in adult non-patients. J Orofac Pain. 2008;22:97–107. 18548838

[24] Saghafi E, Mejersjö C. A method for preventive intervention regarding temporomandibular pain and dysfunction. Acta Odontol Scand. 2018;76:482–487. 10.1080/00016357.2018.1439529 29448878

[25] Hagquist, C. Psychometric properties of the PsychoSomatic problems scale: a Rasch analysis on adolescent data. Soc Indicat Res. 2008;86:511–523. 10.1007/s11205-007-9186-3

[26] Lövgren A, Visscher CM, Häggman-Henrikson B, Lobbezoo F, Marklund S, Wänman A. Validity of three screening questions (3Q/TMD) in relation to the DC/TMD. J Oral Rehabil. 2016;43:729–736. 10.1111/joor.12428 27573533

[27] Kroenke K, Spitzer RL, Williams JB, Löwe B. An ultra-brief screening scale for anxiety and depression: the PHQ-4. Psychosomatics. 2009;50:613–621. 10.1016/S0033-3182(09)70864-3 19996233

[28] Dos Santos EA, Risuenho Peinado BR, Frazão DR, de Sousa Né YG, Fernandes Fagundes NC, Mogno MB, et al. Association between temporomandibular disorders and anxiety: a systematic review. Front Psychiatry. 2022;13:990430. 10.3389/fpsyt.2022.990430 36311527 PMC9606663

[29] John MT, Omara M, Su N, List T, Sekulic S, Häggman-Henriksson B, et al. Recommendations for use and scoring of Oral Health Impact Profile versions. J Evid Based Dent Pract. 2022;22:101619. 10.1016/j.jebdp.2021.101619 35219460 PMC8886153

[30] Branco, L Santis T, Alfaya T, Godoy C, Fragoso Y, Bussadori S. Association between headache and temporomandibular joint disorders in children and adolescents. J Oral Sci. 2013;55:39–43. 10.2334/josnusd.55.39 23485599

[31] Szperka C. Headache in children and adolescents. Continuum (Minneap Minn). 2021;27:703–731. 10.1212/CON.0000000000000993 34048400 PMC9455826

[32] Cho H-J, Park SE, Park JW. Physical activity level and temporomandibular disorders in South Koreans. Community Dent Oral Epidemiol. 2020;48:225–231. 10.1111/cdoe.12519 31994225

